# Large volume headspace GC/MS analysis for the identification of volatile compounds relating to seafood decomposition

**DOI:** 10.1002/fsn3.2751

**Published:** 2022-01-22

**Authors:** Zhengfang Wang, Lowri S. de Jager, Timothy Begley, Susan Genualdi

**Affiliations:** ^1^ Joint Institute for Food Safety and Applied Nutrition University of Maryland College Park Maryland USA; ^2^ Center for Food Safety and Applied Nutrition Office of Regulatory Science U.S. Food and Drug Administration College Park Maryland USA

**Keywords:** large volume headspace GC/MS, pooled calibration curves, seafood, volatile compounds

## Abstract

Decomposition in seafood products in the United States is monitored by the Food and Drug Administration (FDA) laboratories using sensory testing, which requires highly trained analysts. A large‐volume headspace (LVHS) gas chromatography/mass spectrometry (GC/MS) method was developed to generate analytical results that can be directly compared to sensory evaluation. Headspace vapor was withdrawn from a 1‐L vial containing 50 g seafood sample using a large volume headspace autosampler. Various volatile compounds were collected simultaneously. Analytes were preconcentrated by a capillary column trapping system and then sent through a cryo‐focuser mounted onto the GC injector. A selected ion monitoring (SIM) MS acquisition method was used to selectively monitor 38 compounds of interest. Samples of red snapper, croaker, weakfish, mahi‐mahi, black tiger shrimp, yellowfin tuna, and sockeye salmon that have been assessed and scored by an FDA National Seafood Sensory Expert (NSSE) were used for method performance evaluation. Characteristic compounds potentially associated with seafood quality deterioration for each seafood species were identified by quantitative analysis using pooled matrix‐matched calibrations and two‐sample *t*‐test statistical analysis. Classification of fresh and decomposed samples was visualized on the analysis of variance (ANOVA)–principal component analysis (PCA) score plots. The results determined that the LVHS‐GC/MS technique appeared promising as a screening tool to identify compounds representative of sensory analysis.

## Graphical abstract

Large volume headspace GC/MS generated analytical results that can be directly compared to sensory evaluation. Classification of fresh and decomposed seafood samples was successful. VOCs relating to seafood decomposition were identified.

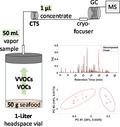

## 1 INTRODUCTION

Fresh seafood is prone to decomposition during transport and storage if there are variations in time and temperature. In the United States, decomposition in seafood products is monitored by the U.S. Food and Drug Administration (FDA) National Seafood Sensory Experts (NSSE), mainly by performing sensory testing (FDA Office of Regulatory Affairs, 2013; Yakes et al., 2021). Since the human olfactory system is extremely sensitive to odor mixtures (McGann, 2017), sensory evaluation with well‐established protocols is reliable, accurate, and sensitive (Food & Agricultural Organization of the United Nations, 1999). However, organoleptic measurements require highly trained analysts. Therefore, there is a need for instrument‐based screening to identify volatile markers using automated devices (Zhang & Li, 2010), which can be used to support FDA NSSE.

Chemical indices of decomposition can provide a significant support mechanism to sensory findings in some seafood products. The FDA has established criteria for analyzing histamine (AOAC Official Method, 1987) and indole (AOAC Official Method, 1982) in seafood to support regulatory action in the absence of sensory evidence in some cases. Other potential chemical indicators of decomposition in specific seafood products may also have utility in determining the decomposed state of seafood (Boziaris & Parlapani, 2017; Joffraud et al., 2001). Several instrumental methods for analyzing chemical indices of seafood freshness have been proposed (Bai et al., 2019; Chan et al., 2006; Duflos et al., 2005; Self et al., 2018). However, these methods always use only a small portion (e.g., 2–10 g) of a whole fillet, which may not accurately represent an entire fish sample. For instance, decomposition caused by oxidation usually occurs in the fish abdomen while bacterial growth would be the main source of deterioration in fish back meat (Khoshnoudi‐Nia & Moosavi‐Nasab, 2019; Wang et al., 2020). Thus, sampling bias may occur when a very small amount of sample is collected from a single region of a fish filet. In addition, advanced chromatography–mass spectrometry techniques often require sample pretreatment, such as extraction or derivatization, to achieve higher sensitivity. But sample pretreatment does not always preserve the original proportion and integrity of volatile compounds, so measurement bias may occur when evaluating the agreement between instrumental analysis and sensory testing. Furthermore, previous studies tend to focus on one or two seafood species and use laboratory‐based samples that were prepared in‐house under controlled conditions.

For these reasons, the present work was undertaken to develop a large volume headspace (LVHS)–gas chromatography/mass spectrometry (GC/MS) method, as a nonsensory analytical technique, for the analysis of seafood decomposition in a way that can be directly compared to FDA NSSE sensory evaluation scores. By using seven types of FDA NSSE graded wild‐caught seafood products at known organoleptic states, the reliability of LVHS‐GC/MS was evaluated. Potential marker compounds indicative of decomposition for each seafood species were identified. Analytical challenges encountered in headspace analysis of volatiles are also discussed.

## 2 MATERIALS AND METHODS

### 2.1 Sample information

Seafood samples used for method development, optimization, and evaluation were fresh and unprocessed Atlantic salmon, tilapia, and cod purchased from local supermarkets in the Washington D.C. area. Samples were stored at −60°C upon arrival at the laboratory.

Seafood samples for investigation were provided by the FDA Pacific Northwest Laboratory in Washington (Self et al., 2018). Frozen, unprocessed fillet portions of seven seafood species (red snapper, croaker, weakfish, mahi‐mahi, black tiger shrimp, yellowfin tuna, and sockeye salmon) were collected from Guyana (red snapper, croaker, and weakfish), Ecuador (mahi‐mahi), Vietnam (black tiger shrimp and yellowfin tuna), and Alaska (sockeye salmon). Each portion was individually evaluated by FDA NSSE near the catch locations, vacuum packed, flash‐frozen, and transported to FDA.

A sensory score on a 100‐point scale was given to each portion by FDA NSSE, where “0” represents the best quality and “100” represents the worst quality. Scores between 0 and 50 were passing (nondecomposed) and scores between 51 and 100 were failing (decomposed). In the current study, samples with sensory scores between 15 and 25 were considered to be “fresh”; samples with sensory scores higher than 70 were considered to be “decomposed.” Sensory scores and odor characteristics are described in Table 1.

**TABLE 1:** Sensory description for seafood samples used in this study

Species	Country of origin	Sensory description			
		Pass (fresh)		Fail (decomposed)	
		Sensory score	FDA NSSE comments	Sensory score	FDA NSSE comments
Red snapper	Guyana	20–25	Citrus	>70	Yeasty
Croaker	Guyana	20–23	Pondy	>70	Garbage
Weakfish	Guyana	20–26	Briny and neutral	68–75	Fermented and sour
Mahi‐mahi	Ecuador	15–20	Not available	>75	Not available
Black tiger shrimp	Vietnam	15–25	Sweet and neutral	>70	Sour
Yellowfin tuna	Vietnam	20–25	Slightly meaty and metallic	>75	Fermented and putrid
Sockeye salmon	USA/Alaska	23	Slight sweet	>70	Sour

### 2.2 Chemicals

The chemical standards dimethyl sulfide, carbon disulfide, 2‐methyl‐1‐propanal, 2,3‐butanedione, 2‐butanone, chloroform, 2‐methyl‐1‐propanol, 3‐methylbutanal, 3‐methyl‐2‐butanone, 2‐methylbutanal, 2‐pentanone, 1‐penten‐3‐ol, pentanal, 3‐pentanone, acetoin, 3‐methyl‐1‐butanol, methylcyclohexane, dimethyl disulfide, 2‐methyl‐1‐butanol, 2‐penten‐1‐ol, 3‐hexanone, hexanal, ethyl butyrate, 1,2‐dimethylcyclohexane, 1‐hexanol, 2‐heptanone, dimethyl trisulfide, octanal, 2‐ethyl‐1‐hexanol, 2‐nonanone, nonanal, decanal, 2‐undecanone, and 4‐heptanone were purchased from Sigma‐Aldrich. The chemical standards 2,4‐octadiene and 1,1,3‐trimethylcyclohexane were purchased from BOC Sciences. Pentane, n‐heptane, toluene, sodium sulfate (Na_2_SO_4_), and anhydrous powder (ACS grade) were purchased from Fisher Scientific.

### 2.3 Sample preparation

All seafood samples were individually vacuum sealed and kept frozen at −60°C prior to use. In the Figure S1 shows that a typical fish filet in this study included both back and abdomen regions. Once a deep‐frozen sample was slightly thawed, inedible skin or shell was removed. The edible parts were placed in a Robot Coupe R401B single‐speed food processor and ground for 1 min while still mostly frozen. Aliquots of the ground seafood (50.0 ± 0.1 g) and drying agent Na_2_SO_4_ (35.0 ± 0.1 g) were placed in a 1‐L glass headspace vial. Figure S1A is a picture of the headspace vial cap equipped with the Bottle‐Vac™ O‐ring seal. The number of biological replicates for each type of seafood depended on the availability of the seafood samples under analysis.

### 2.4 Headspace sampling

Prior to headspace sampling, each 1‐L headspace vial was incubated at 30°C for 30 min with agitation. After incubation, an aliquot of headspace vapor (50 ml) was withdrawn directly from the 1‐L vial through the Silonite™ Male Micro‐QT™ valve mounted on the top of the vial cap (Figure S1C).

Collected headspace vapor went through a capillary column trapping system (CTS) to eliminate water and air. Vapor sample size was then reduced from 50 ml to 1 µl. Concentrated analytes went through a cryo‐focuser mounted onto GC injector. Liquid nitrogen focused the analytes onto the GC column (Wilson et al., 2012; Wylie, 1986), which significantly improved peak shape and resolution.

The entire headspace sampling process was automatically performed using a 7650HS‐CTS analyzer (Entech Instruments). Optimization of experimental parameters can be found in the Figures S2–S5 and Table S1 and S2. This headspace autosampler system has options for static sampling, CTS trapping, cryo‐focusing, and injection. Key parameters were provided in Table S2.

### 2.5 GC/MS analysis

GC/MS analysis of volatile compounds was performed using an Agilent 7890B gas chromatograph/5975C single quadrupole mass spectrometer with a DB‐1ms column (60 m × 0.32 mm i.d. × 0.25 μm film thickness). The GC temperature program was as follows: 27°C, hold for 7 min; ramp at 15°C/min to 250°C; ramp at 100°C/min to 300°C, hold for 5 min. The injector temperature was 260°C. The carrier gas (helium, 99.99% purity) flow rate was 1 ml/min constantly. The solvent cut time was 5.8 min. The interface temperature was 260°C. The MS ion source temperature was 250°C. The MS quadrupole temperature was 180°C. The EI energy was 70 eV.

Although many flavor compounds are polar, several target compounds in our study are water insoluble, including 2‐methyl‐1‐propanal, 2‐nonanone, nonanal, decanal, and 2‐undecanone. In order to obtain good separation of all of the target compounds in different species, a DB‐1ms column (60 m × 0.32 mm × 0.25 μm) was used. The 0.32 mm i.d. increased sample capacity, which allowed for the simultaneous measurement of analytes at a wide range of concentrations and the 60 m column length improved resolution.

### 2.6 Identification of volatile compounds

GC/MS data were collected using MassHunter GC/MS Acquisition B.07.03.2129 software (Agilent Technologies). The identity of each target compound was determined by comparing its retention time, major ions, and isotopic pattern with an analytical standard analyzed under the same conditions in SCAN mode. Then, the selected ion monitoring (SIM) time segments were created using unique and abundant ion fragments of each target compound.

### 2.7 Interference removal

Excessive water in the headspace above seafood samples was reduced using Na_2_SO_4_ anhydrous powder to prevent chromatographic distortion and mass spectrometric interference. In addition to moisture, interferences due to the prevalence of volatile organic compounds in the laboratory can be problematic in headspace analysis. Reagents, glassware, and other sampling hardware may also yield artifacts to sample analysis. Thus, steps were taken to reduce interfering compounds.

In this study, all glass vials were baked at 150°C overnight before use. Lids, valves, and O‐rings were stored in a vacuum oven. A SIM MS acquisition method was used to acquire signals at only the selected mass fragments in a certain time segment. Moreover, baseline correction, a chemometric data preprocessing method, was used to further remove interfering and irrelevant signals from analytical signals (Wang et al., 2014). A blank (i.e., headspace vial only containing the drying agent Na_2_SO_4_) was run in the same manner as seafood samples. A total of 30 blanks were obtained. Two‐way (chromatographic and mass spectrometric) baseline correction was performed with an in‐house algorithm after data normalization (Wang et al., 2013).

### 2.8 Calibration curves

A calibration mixture stock solution was prepared with 38 chemical standards listed in Table 2. Calibration solutions were made by serial dilutions of the stock solution with pentane to create multiple concentration levels. The internal standard solution was 4‐heptanone (653 μg/kg in pentane).

**TABLE 2:** Performance of pooled matrix‐matched calibration curves

	Compounds	Odor^a^	Pooled matrix‐matched calibration curves			Cod spikes (*n* = 3)			
			*R* ^2^	Lowest conc. (ppb)	Highest conc. (ppb)	Spiked conc. (ppb)	Calculated conc.		Accuracy^c^
							Average	%RSD^b^	
Very Volatile Organic Compounds (VVOC)	2,3‐Butanedione	Strong, chlorine‐like	0.9636	283	18,585	1056	1103	22%	104%
	2‐Butanone	Sweet, acetone‐like	0.9515	13	4620	236	361	22%	153%
	2‐Methylbutanal	Cocoa or coffee‐like	0.9691	47	17,478	426	432	29%	101%
	3‐Methyl−2‐butanone	Pleasant, acetone‐like	0.9849	48	29,167	885	1458	22%	165%
	3‐Methylbutanal	Penetrating, apple‐like	0.9723	117	14,547	434	668	18%	154%
	Dimethyl sulfide	Wild radish, cabbage‐like	0.9711	19	1376	70	64	29%	91%
	Carbon disulfide	Sweet, ether‐like	0.9727	4	2294	70	81	17%	117%
	Chloroform	Pleasant, etheric	0.9728	5	2159	83	107	31%	129%
	Heptane	Petroleum‐like	0.9755	41	24,783	755	991	35%	131%
Volatile Organic Compounds (VOC)	1,1,3‐Trimethylcyclohexane	Pungent acrid	0.9770	2	746	42	63	17%	149%
	1,2‐Dimethylcyclohexane	Mild characteristic	0.9800	23	14,094	425	627	17%	148%
	1‐Hexanol	Characteristic, sweet alcohol, pleasant	0.9859	25	8950	452	498	21%	110%
	1‐Penten−3‐ol	Powerful, grassy‐green	0.9707	50	15,199	917	1116	19%	122%
	2,4‐Octadiene	Tropical fruit, grapefruit‐like	0.9623	24	7617	433	866	16%	200%
	2‐Ethyl−1‐hexanol	Mild, oily, sweet, slightly floral	0.9751	5	1627	93	115	51%	125%
	2‐Heptanone	Penetrating banana‐like	0.9673	3	1486	145	95	9%	66%
	2‐Methyl−1‐butanol	Wine‐like or onion‐like	0.9768	49	20,382	900	1268	14%	141%
	2‐Methyl−1‐propanal	Extremely sharp, pungent	0.9779	23	14,312	427	457	7%	106%
	2‐Methyl−1‐propanol	Sweet, musty	0.9742	60	7274	224	232	19%	104%
	2‐Nonanone	Fruity, floral, fatty, herbaceous	0.9203	3	1188	46	48	35%	106%
	2‐Pentanone	Characteristic acetone‐like	0.9807	2	1173	88	87	7%	99%
	2‐Penten−1‐ol, (E)‐	Ethereal, fruity	0.9785	25	15,453	1052	961	10%	91%
	2‐Penten−1‐ol, (Z)‐	Ethereal, fruity	0.9868	237	7898	452	415	7%	92%
	2‐Undecanone	Citrus, fatty, rue‐like	0.9595	90	1587	90	133	29%	148%
	3‐Methyl−1‐butanol	Disagreeable, choking	0.9741	49	10,644	889	868	6%	98%
	3‐Hexanone	Sweet, fruity, waxy, diffusive	0.9766	24	14,765	446	833	13%	187%
	3‐Pentanone	Acetone‐like	0.9703	12	7364	224	330	22%	147%
	Decanal	Fatty, floral‐orange	0.9538	5	1298	91	105	1%	116%
	Dimethyl disulfide	Garlic‐like	0.9809	3	1737	158	234	13%	148%
	Dimethyl trisulfide	Characteristic, garlic‐like, sulfurous	0.6484	3	2017	58	ND		
	Ethyl butyrate	Pineapple‐like	0.9008	49	15,840	1900	2284	23%	120%
	Methylcyclohexane	Faint, benzene‐like	0.9728	5	2790	86	101	26%	118%
	Hexanal	Characteristic fruity, green grass	0.9758	12	7382	58	59	33%	101%
	Pentanal	Strong, acrid, pungent	0.9731	48	35,504	881	998	17%	113%
	Nonanal	Rose‐orange, floral, waxy, green	0.9752	5	1496	90	94	1%	104%
	Octanal	Strong, citrus‐like	0.9718	5	1485	47	30	10%	65%
	Toluene	Sweet, pungent, benzene‐like	0.9816	3	1569	48	78	24%	163%

^a^ Description of odor was found on the PubMed^®^ database.

^b^ The percent accuracy was the calculated concentration of each compound divided by its expected concentration in cod spike, expressed in units of percent.

^c^ %RSD stands for percent relative standard deviation.

During this research, two types of calibration curves were prepared. The first type of calibration curve was made by adding 10 µl of each calibration solution and 10 µl of internal standard solution onto a piece of blank Whatman^®^ qualitative filter paper (Grade 1, 85 mm circle) and the second type was pooled matrix‐matched calibration curves generated using 50 g of ground tilapia (along with 35 g of Na_2_SO_4_ powder) spiked with 10 µl of each calibration solution.

Filter paper was initially used as a blank to make calibration standards because nonpolar chemical standards are insoluble in water and to avoid potential interferences from solvents. Only a very small amount of calibration solution (10 µl) was spiked into each calibration standard for the purpose of minimizing solvent (pentane) vapor in the headspace glass vial. Details of concentrations of individual analytes in each calibration level can be found in Table S3. Precision and accuracy of filter paper‐based calibration curves were assessed using three biological replicates of blank (filter paper) spikes.

For the pooled matrix‐matched calibration curves, tilapia was chosen to be the matrix because its fat content was between the low and high range of other types of seafood to be analyzed (Genualdi et al., 2013). Five sets of calibration curves were individually made on five different days. Concentration levels varied by analyte (Table 2). A regression line of best fit was generated from the pooled data of all calibrations combined so day‐to‐day variation and matrix effects were factored into the curves. Peak area measurement, generation of calibration curves, and concentration calculation were performed by MS Quantitative Analysis, version 10.2 (Agilent Technologies). Calibration curves shown in Figure S6 were reconstructed using Excel, version 2102 (Microsoft). The established pooled matrix‐matched calibration curves were assessed using three biological replicates of matrix spikes. A different matrix (cod) was used to evaluate the pooled matrix‐matched calibration curve made with tilapia. Cod as a very lean fish is low in fat and carbohydrates. Blank fresh cod typically had a small amount, if any, of the analytes detectable prior to spiking. Matrix spikes were prepared with 50 g of cod (along with 35 g of Na_2_SO_4_ powder) spiked with 10 µl of a spike solution. Blank subtraction was performed prior to quantification.

### 2.9 Statistical calculation

Baseline correction (Wang et al., 2013) and analysis of variance (ANOVA)–principal component analysis (PCA) (Harrington et al., 2005) were performed using MATLAB R2021b (MathWorks). Raw GC/MS data sets were acquired as network common data form (netCDF) files. With an in‐house algorithm, netCDF files were read into MATLAB. The data sets were binned by retention time from 5.8 min to 21.0 min with a 0.01 min increment and binned by mass‐to‐charge ratio from *m/z* 22 to *m/z* 175 with a *m/z* 0.1 increment. Data sets were normalized to unit vector length to reduce random errors, such as slightly varying amounts of samples in different injections. After baseline correction, the separability of data clusters was visualized on ANOVA‐PCA score plots.

Concentrations of target compounds in fresh and decomposed seafood samples were subject to two‐sample *t*‐tests to determine potential marker compounds indicative of decomposition for each species. A two‐sample *t*‐test was performed using the *ttest2* function in MATLAB, which returned the values of average, standard deviation (STD), and *h*. The *h* value is a test decision for the null hypothesis that two groups of data come from independent random samples from normal distributions with equal means. The result *h* is 1 if the test rejects the null hypothesis at the 5% significance level, and 0 otherwise. Thus, a compound with a *h* value of 1 was considered to be a potential marker compound.

## 3 RESULTS AND DISCUSSION

### 3.1 Identification of volatile compounds in seafoods

Seafood is diverse in many ways. The present study included seven seafood species caught from various habitats. To discover marker compounds relating to quality deterioration in seafood, initially a nontargeted fingerprinting method using GC/MS data sets collected in the SCAN mode was attempted (data not shown). However, a high number of unspecified data points from interfering compounds limited the possibility of making one group of samples strikingly different from other groups. Thus, a targeted method was developed herein. Targeted analysis often has a higher selectivity and sensitivity than nontargeted analysis. Moreover, the reliability of a targeted method can be validated using chemical standards, as analytical targets have been predefined (Ballin & Laursen, 2019).

The headspace of seafood samples contains a variety of volatile compounds. These compounds cover a wide range of physical and chemical properties. In this study, 38 compounds of interest were investigated for the spoilage of seafood. Their odor characteristics are provided in Table 2. Among them, nine compounds with a boiling point lower than 100°C were categorized as very volatile organic compounds (VVOC) and the other 29 compounds are volatile organic compounds (VOC; EURO Reports & Studies 111, 1989). Volatile amines, a traditional chemical indicator of seafood spoilage, were excluded herein because they are generally known to have a basic character (Chung & Chan, 2009; Monique, Ifremer, & Nantes, 2005; Teixeira et al., 2019) and will be the focus of future studies.

All the 38 target compounds in the current study have been reported in other literature as being correlated with quality change in various types of food (Alasalvar et al., 2005; Bai et al., 2019; Duflos et al., 2005; Joffraud et al., 2001; Jørgensen & Henrik, 2001). The primary goal of the current study is to establish and evaluate a new large volume headspace sampling method for detecting these target compounds in seafood and identify compounds that have significant differences in concentration between fresh and decomposed seafood.

Fish spoilage usually results from three mechanisms: enzymatic autolysis, oxidation, and microbial growth (Ghaly et al., 2010; Takahashi et al., 2004; Tavares et al., 2021; Varlet & Fernandez, 2010). Sources of certain compounds have been fully investigated. For example, 1‐hexanol, 1‐penten‐3‐ol, 2,3‐butanedione, 2‐ethyl‐1‐hexanol, 2‐heptanone, 2‐methyl‐1‐propanol, 2‐methylbutanal, 2‐pentanone, 2‐penten‐1‐ol, 3‐methyl‐1‐butanol, 3‐methyl‐2‐butanone, 3‐methylbutanal, 3‐hexanone, 3‐pentanone, and acetoin can originate from main microbial catabolic pathways of lipids, carbohydrates, and amino acids (Boziaris & Parlapani, 2017; Joffraud et al., 2001; Jørgensen & Henrik, 2001). Sulfur‐containing volatiles, such as dimethyl sulfide, dimethyl disulfide, dimethyl trisulfide, and carbon disulfide, can be produced by microbial‐mediated enzymatic reaction, mainly degradation of amino acids (Varlet & Fernandez, 2010). Even in vacuum packed seafood during storage, spoilage bacteria still could generate volatile compounds, such as 2‐methyl‐1‐butanol and 2‐butanone (Jørgensen & Henrik, 2001). Some low molecular weight compounds are primary and secondary lipid oxidation products with strong olfactory attributes, imparting the characteristic odor of rancid fish oil (Kulås et al., 2002), including 2,4‐octadiene, 2‐methyl‐1‐propanal, 2‐nonanone, 2‐undecanone, decanal, ethyl butyrate, hexanal, nonanal, octanal, and pentanal. Multiple artifacts have been found in seafood during storage, but their occurrence does not contribute to the development of decomposed seafood odor, such as toluene, heptane, and chloroform (Alasalvar et al., 2005; Bai et al., 2019). Origins of saturated hydrocarbons in seafood, including 1,1,3‐trimethyl‐cyclohexane, 1,2‐dimethyl‐cyclohexane, and methylcyclohexane, are not fully known, but their occurrence in various seafood has been reported by other researchers (Duflos et al., 2005; Shimoda et al., 1996).

### 3.2 Large volume headspace sampling optimization

Large volume headspace sampling was optimized for equilibration time, withdrawn volume of vapor, agitation, and addition of sample modifiers. Details on the results of the optimization can be found in the Supplemental Material. To make the instrumental technique potentially a more fitting companion tool to sensory testing, the incubation temperature (30°C) was set to be slightly above ambient temperatures (27 ± 3°C). At 30°C, a 30‐min incubation with agitation was found to be sufficient to allow the concentrations of headspace compounds in a 1‐L glass vial to reach apparently steady values. A comparison of different equilibration time also showed that a prolonged incubation period did not promote the mass transfer across the phase boundary (Figure S2) but extended the sample run time (Table S1). Agitation during incubation proved to be advantageous to help less volatile compounds diffuse in the headspace phase and be effectively withdrawn from the vial (Figure S3).

In many cases, the addition of salts or solvents to sample matrix may decrease the partition coefficients and allow more compounds to pass into the headspace phase. Three different types of matrix modifier were tested during method development: saturated sodium chloride (NaCl) water, 10% potassium hydroxide (KOH) water, and Na_2_SO_4_ anhydrous powder. The addition of NaCl solution to aqueous samples did not result in an observable difference for the current study of seafood. Adding 10% KOH to ground seafood, in an attempt to breakdown fat and lipids, was not successful in this study because saponification did not occur at the current incubation temperature of 30°C. Instead, the presence of alkali aqueous solution has changed the original volatile composition of ground seafood. For example, ethyl butyrate and 2,3‐butanedione reacted with alkali in water. By contrast, adding Na_2_SO_4_ as a drying agent to ground seafood increased the headspace concentrations of most compounds, implying that removing moisture from headspace vapor improved the transfer of volatiles to the headspace (Figure S4).

After volatile compounds in the 1‐L vial reached equilibrium, a portion of the headspace vapor was withdrawn from the vial in a nonselective manner: various analytes were collected along with water and air. A desirable volume to withdraw from the headspace was found to be 50 ml, since a smaller volume would not allow the effective collection of less volatile compounds. Withdrawing a larger volume of headspace vapor, by contrast, did not appear to have a significant effect on the detection limits of most compounds (Figure S5) but resulted in poor peak shapes of early eluting compounds in GC chromatograms. Withdrawing a large volume of headspace vapor would also drastically prolong the headspace sampling process (Table S1).

It should be noted that the large volume headspace sampling method is different from traditional headspace techniques, such as solid‐phase microextraction (SPME) and purge‐and‐trap. There are several advantages of the large volume headspace sampling method, including (1) reduced sampling bias. Since a 1‐L headspace vial has a higher capacity than a regular headspace vial (6–27 ml), different regions of a whole fillet were tested in a single analysis. Also, (2) the overall pattern of VVOCs and VOCs in seafood headspace was not altered during sample preparation because a minimal sample pretreatment protocol was used. Moreover, (3) a variety of aroma compounds were recovered at the same distribution as experienced by the sensory analyst. As the headspace vapor collection process was nonselective, compounds generated from different sources were extracted in the same manner.

### 3.3 PCA analysis with data generated by LVHS‐GC/MS

**FIGURE 1:**
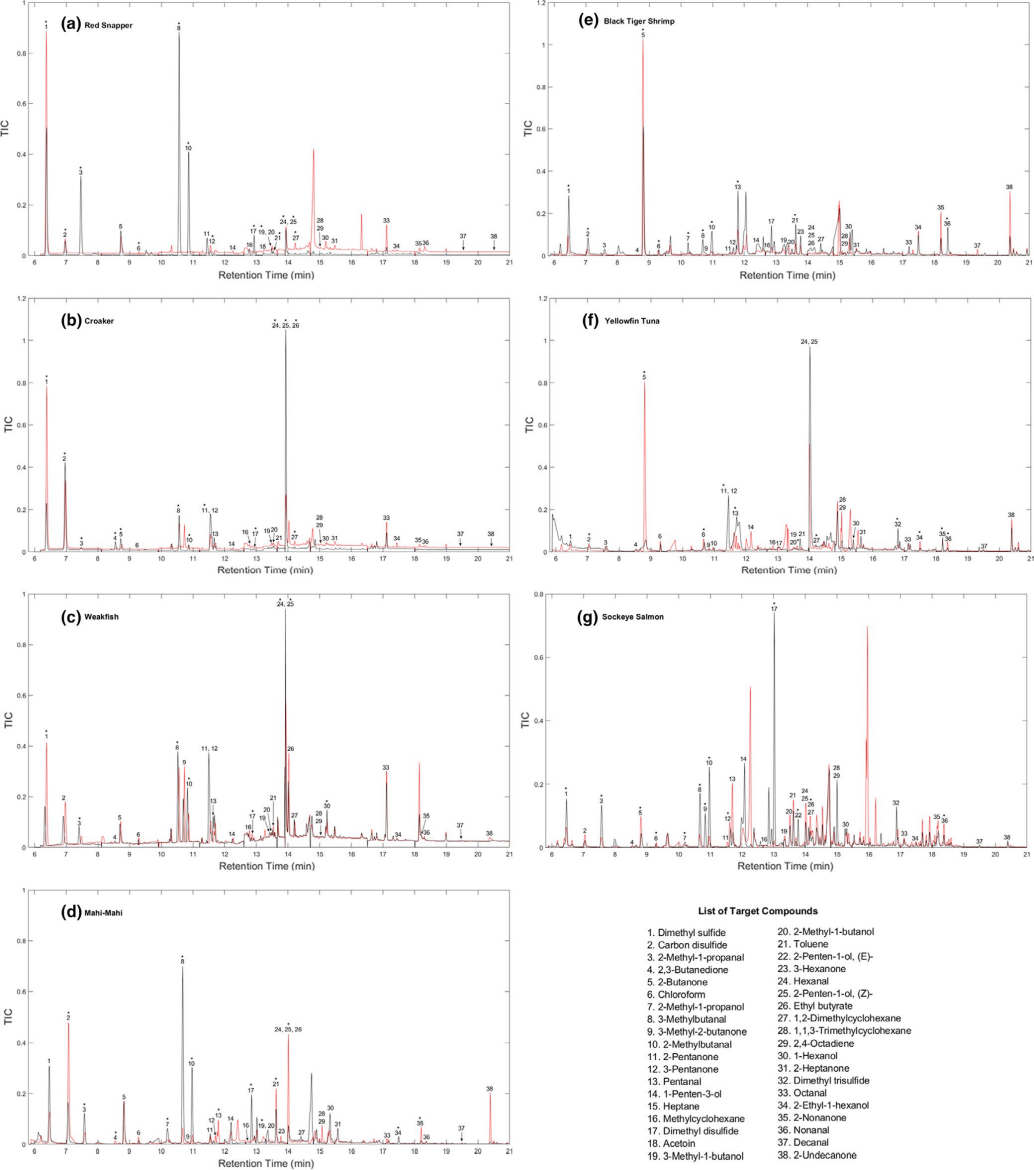
Normalized TICs of fresh (red line) and decomposed (black line) seafood samples: (a) Red snapper, (b) croaker, (c) weakfish, (d) mahi‐mahi, (e) black tiger shrimp, (f) sockeye salmon, and (g) yellowfin tuna. Chemical profiles were collected using the established LVHS‐GC/MS method. Potential marker compounds relating to seafood decomposition (with the *h* value of 1 for the two‐sample *t*‐test by MATLAB) were marked with a * sign

Seafood freshness assessment using LVHS‐GC/MS chemical profiles was directly compared with sensory scores obtained by FDA NSSE. The established LVHS‐GC/MS method proved to be effective in differentiating fresh and decomposed seafood samples of red snapper, croaker, weakfish, mahi‐mahi, black tiger shrimp, yellowfin tuna, and sockeye salmon. For each species, total ion chromatograms (TIC) of one fresh sample and one decomposed sample are provided as examples in Figure 1. Results show that different compounds were produced in each seafood species during decomposition so different types of seafood could have distinct chemical indices of decomposition. Peaks corresponding to target compounds were denoted. Each compound was positively identified using a corresponding analytical standard.

**FIGURE 2:**
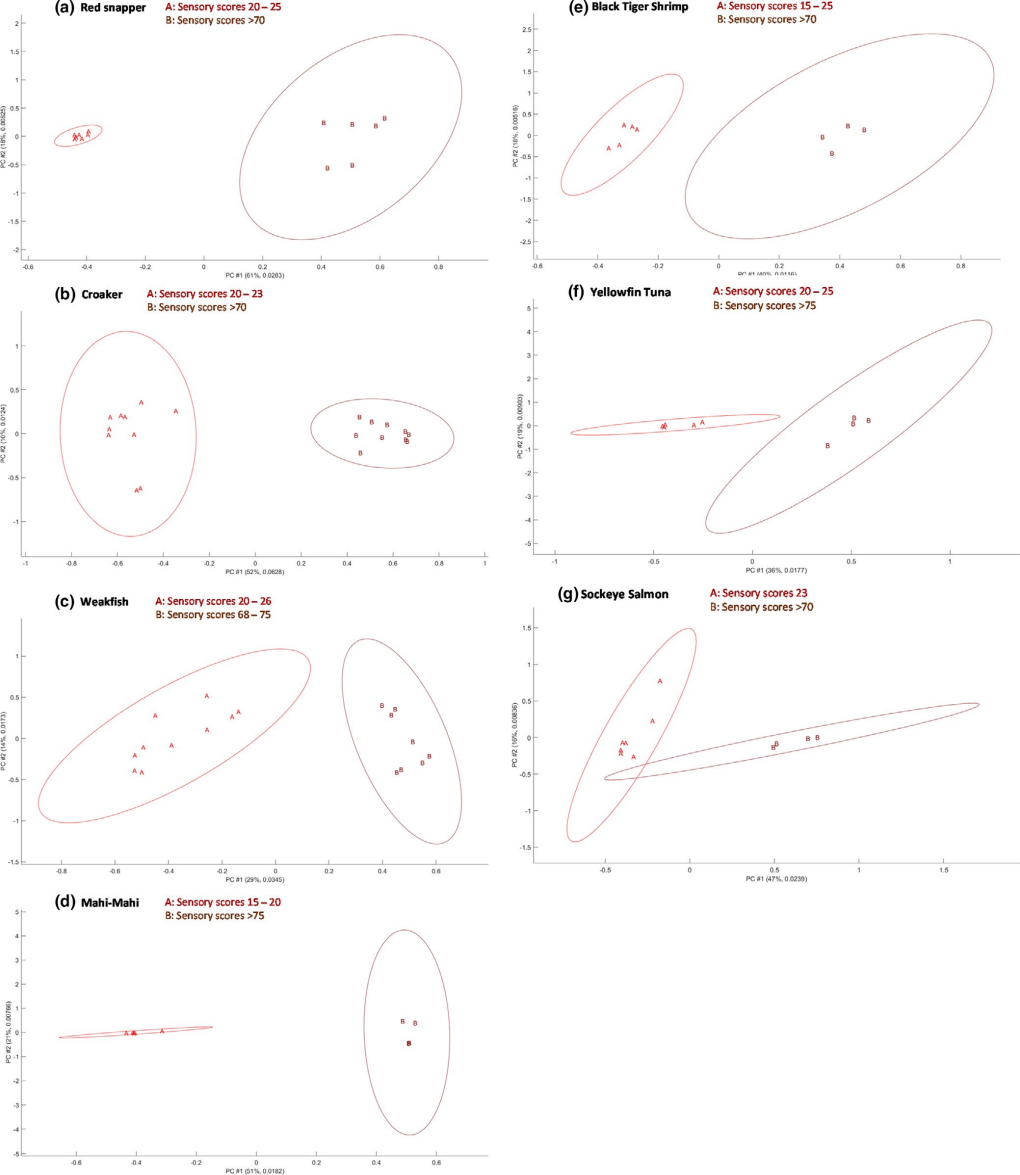
Separability of the LVHS‐GC/MS data sets of fresh and decomposed (a) red snapper, (b) croaker, (c) weakfish, (d) mahi‐mahi, (e) black tiger shrimp, (f) sockeye salmon, and (g) yellowfin tuna samples on the ANOVA‐PCA score plots. Ovals are 95% confidence intervals. All samples have been previously evaluated by FDA NSSE. Group A was fresh samples. Group B was decomposed samples. Sample size depended on the availability of the seafood samples under analysis

Complex GC/MS data sets were interpreted using a chemometric data analysis strategy. PCA converted GC/MS data sets to a lower‐dimensional space without any awareness of the class labels. The ANOVA‐PCA score plots were used to visualize the separation of data clusters. As shown in Figure 2, for red snapper, croaker, weakfish, mahi‐mahi, black tiger shrimp, and yellowfin tuna, fresh and decomposed samples were completely separated. Sample classification based on LVHS‐GC/MS analysis agreed well with FDA NSSE sensory analysis. For sockeye salmons, 95% confidence intervals of two data clusters overlapped to some extent, indicating that profiling volatile compounds of interest may not be sufficient to accurately assess the freshness of sockeye salmon.

The major obstacle to accurate classification of fresh and decomposed seafood samples using instrumental analysis was the large variability among multiple samples of the same type. Essentially, in the present study, each sample was representative of an individual fish so biological replicates represented different fish with a sensory score that fell within a certain range. Therefore, the data variability was greater than if one fillet was homogenized and separated into multiple samples for replicates.

The large data variance was observed on PCA score plots as wide 95% confidence intervals. In PCA, a primary set of data sets can be represented by a subset of independent principal components (PCs) (Gniazdowski, 2017) such that new data sets typically contain fewer variables than the original ones. Ideally, a small number of principal components can contain as much information as the full set of primary variables. For example, Figure 2a shows that the percentage of variance explained by the first and the second PCs was 61% and 18%, respectively. Their cumulative percentage of variance was 79%. It means that the first two principal components carryover 79% of the information contained in primary variables. It should be noted that PCA was used to observe the clustering of LVHS‐GC/MS data sets. Cohesion and separation of data clusters can be improved if a higher number of the extracted PCs were used (e.g., four PCs). But choosing a subset of principal components seems slightly out of scope and will not be discussed herein. The large data variance implied that marker compounds could potentially be better characterized by quantitative analysis.

### 3.4 Quantitative analysis with LVHS‐GC/MS

Acquiring quantitative data can be challenging with GC headspace analysis, especially for the analysis of natural products in which multiple compounds are present with different chemical properties at various concentrations. Accurate quantification of VVOCs and VOCs is more difficult, considering the volatility and the possible biases caused by unknown partition behavior, the variety of polar and nonpolar compounds, and the unavailability of blank matrices.

To confirm the LVHS‐GC/MS procedure is suitable for quantitative analysis of volatiles, filter paper‐based calibration curves were assessed using three biological replicates of blank (filter paper) spikes. Precision in this study was determined as the repeatability relative standard deviation (%RSD) of three spikes. The percent accuracy was the calculated concentration of each compound divided by its expected concentration in the spike, expressed in units of percent. Due to common coelution and retention time shift of peaks, a qualifier ion has been chosen for each compound in addition to a target ion. The extracted ion chromatogram for the target ion was used for the quantitation, while the qualifier ion was used to facilitate distinguishing this compound from any others with similar retention times.

Partition coefficients of analytes in filter paper‐based calibration standards were free of matrix effects. Looking at just the peak area responses, under the same experimental conditions, %RSD of three blank spikes ranged from 2% to 22%, with 26 of 37 compounds having %RSDs <10%; and accuracy ranged from 78% to 112%, with 24 of 37 compounds having accuracy in the range of 90% and 110%. Quantitation of acetoin was not achieved in this study because extraction followed by direct injection GC/MS (Pinu & Villas‐Boas, 2017) or derivatization followed by headspace GC/MS (Tian et al., 2009) is usually necessary for sensitive detection of acetoin. Result shows that the precision and accuracy of filter paper spikes were satisfactory (Table S3).

However, it was found that target compounds in cod spikes had extremely low peak area responses, indicating that the matrix affected the partitioning of analytes from seafood into the headspace. It is important to consider the variability of the matrix due to the physiological nature of the sample during the implementation of a method (Foods Program Regulatory Science Steering Committee, 2019). Matrix‐matched calibration curves were necessary for the quantitative analysis of volatiles in seafood. Traditional matrix‐matched calibration curves made by taking three replicate measurements at five concentration levels were not satisfactory because the precision of matrix spikes was not adequate for ensuring proper calibration curves (data not shown). A pooled matrix‐matched calibrations approach was adopted to reduce bias (Andersen et al., 2012). The idea was to independently perform measurements of matrix‐matched calibrations at different concentration levels on multiple days and to obtain the line of best fit from the pooled data of all calibrations combined. A larger number of biological replicates were made at low concentration levels than at high concentration levels because low concentration points are usually less precise than high concentration points. Initially, an internal standard (4‐heptanone) was used in the pooled matrix‐matched calibration curves but was later removed because it was not effective at improving the accuracy of the pooled matrix‐matched calibration curves.

**FIGURE 3:**
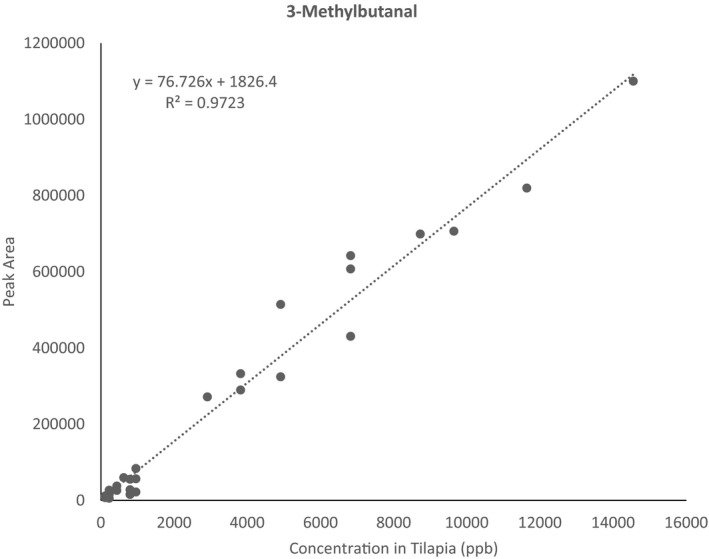
Pooled matrix‐matched calibration curve of 3‐methylbutanal. Concentration levels were 117, 228, 434, 623, 796, 956, 2909, 3819, 4911, 6823, 8728, 9646, 11,638, and 14,547 µg/kg in tilapia. Data points were collected on five different days

In this study, pooled matrix‐matched calibrations had wide linear ranges and linear regression spanned 1–70 orders of magnitude, depending on the analyte (Table 2). It indicates that LVHS‐GC/MS has a very high upper limit of saturation. A pooled matrix‐matched calibration curve of 3‐methylbutanal was provided in Figure 3 as an example. Curves for the rest of the analytes can be found in Figure S6.

Precision and accuracy of the pooled matrix‐matched calibration curves were evaluated using three replicates of spiked cod. Depending on the analyte, the concentration in the spiked cod was at least two times higher than the lowest detectable concentration in blank cod. Blank subtraction was conducted prior to quantification. As shown in Table 2, for pooled matrix‐matched calibration curves, %RSD of three matrix spikes ranged from 1% to 51%, with 20 of 37 analytes having %RSDs less than 20%; and accuracy ranged from 65% to 200%, with 22 of 37 compounds having accuracy in the range of 70% and 130%. This result suggests that pooled matrix‐matched calibrations could correct the influence of matrix to some extent and improve accuracy, although multiple internal standards matching different analyte classes would be needed to improve the accuracy of all target compounds. Since the focus of this study was to identify markers of decomposition, the pooled matrix‐matched calibrations were sufficient to distinguish between fresh and decomposed seafood samples.

The concentrations of 37 analytes in red snapper, croaker, weakfish, mahi‐mahi, black tiger shrimp, yellowfin tuna, and sockeye salmon samples with both passing and failing sensory scores were calculated using the pooled matrix‐matched calibration curves. Quantitative data of marker compounds for seven seafood species are provided in Table S4. Two observed sources of data variability in the preset study were (1) biological variation caused by the fact that each biological replicate represented an individual wild‐caught seafood sample, and (2) noncontrollable variation, as it is impossible to strictly control the VVOC and VOC emissions from 50 g of ground sample into a 1‐L headspace. Even with this variability, differences in headspace concentrations of certain compounds caused by decomposition were still noticeable.

### 3.5 Determination of chemical markers for each species

**FIGURE 4:**
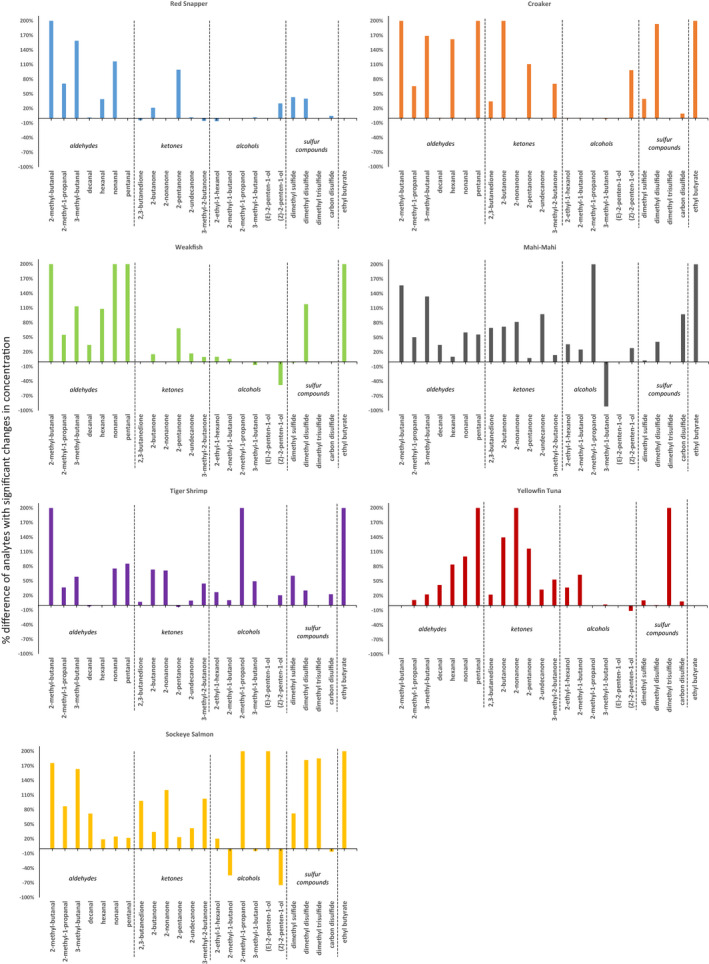
The change in concentrations of important marker compounds for each seafood species. *Y*‐axis is defined as $\frac{{\text{Conc}}_{\text{decomposed}}‐{\text{Conc}}_{\text{fresh}}}{\text{Average}\;\text{Conc}.}\times 100\%$

The off‐odors and off‐flavors of unprocessed seafood are dependent on seafood species and origin (Whitfield, 1998). Statistical tests were performed to determine which target compound could be possible markers relating to decomposition of each species. For each target compound, its concentrations in multiple biological replicates of fresh and decomposed samples were provided in Table S4. Two‐sample *t*‐test determined whether the “fresh” and the “decomposed” populations were statistically different from each other. The requirements of two‐sample *t*‐test were met, since (1) data in each group were obtained via a random sample from the population, (2) data values within a group were independent, (3) the measurements were continuous, and (4) variances for the two groups were assumed to be equal. The target compounds with significant differences in concentration between fresh and decomposed samples are displayed in Figure 4 for each seafood type and by compound class.

#### 3.5.1 Aldehydes

Aldehydes have been confirmed to be important volatile compounds in many different types of seafood (Bai et al., 2019; Duflos et al., 2005; Fratini et al., 2012; Joffraud et al., 2001; Jørgensen & Henrik, 2001). Saturated linear aldehydes (decanal, hexanal, nonanal, and pentanal) and branched‐chain aldehydes (2‐methylbutanal, 2‐methylpropanal, and 3‐methylbutanal) can be generated by different mechanisms. Chemical formation of aldehydes often occurs in heat‐treated products, while in fresh seafood, aldehydes are formed mainly by enzymatic or microbial oxidation reactions of fatty acids (Cserháti & Forgács, 2003; Zhang et al., 2020). The terminal carbonyl group can make an aldehyde relatively reactive and be easily reduced to alcohols or oxidized to acids, and thus the concentrations of aldehydes in seafood are often low (Smit et al., 2009). However, as secondary oxidation products having very low odor thresholds, aldehydes can strongly stimulate olfactory bulbs (Cometto‐Muñiz et al., 1998; Takahashi et al., 2004), so aldehydes can contribute to the characteristic odors determining the sensory quality of seafood.

Our results show that 2‐methylbutanal and 3‐methylbutanal had large percent differences in concentration between fresh and decomposed samples in all species except yellowfin tuna and pentanal was an important marker for yellowfin tuna, weakfish, and croaker. Table S4 shows that different aldehydes were positively correlated with the spoilage of different species (*h* = 1). Monitoring aldehydes could be useful for assessing the freshness of seafood in the future. Especially, 3‐methylbutanal, an intermediate in the catabolism of leucine (Cserháti & Forgács, 2003), was the only significant marker compound found in all seven seafood species. This short‐chain branched aldehyde is an important flavor compound in various foods. Its biochemical conversion routes have been thoroughly reviewed by other researchers (Smit et al., 2009). One possibility is that during the degradation of unprocessed seafood, leucine is liberated from protein by extracellular and intracellular proteolysis; then, multiple transaminases and leucine dehydrogenase catalyzed the conversion of leucine toward α‐keto isocaproic acid; lastly, the branched‐chain α‐ketoacid dehydrogenase complex catalyzed the decarboxylation of α‐keto isocaproic acid to form 3‐methylbutanal. Another option for heat‐treated seafood products is that 3‐methylbutanal can be produced by nonenzymic Strecker degradation. An ideal marker would be one of microbial degradation. Since 3‐methylbutanal is a chemical indicator of protein degradation and can also be produced as a storage odor (Duflos et al., 2005; Jónsdóttir et al., 2008; Smit et al., 2009), further studies are needed to determine how effective of a marker it would be for seafood decomposition.

#### 3.5.2 Ketones

Ketones are another class of volatiles closely related to distinct flavors of seafood. They can be produced by lipid peroxidation. The primary products of lipid oxidation, such as conjugated dienes and hydroperoxides, may be indicators of the initial stage of oxidative degradation (Zhou et al., 2020). In the current study, however, 2,4‐octadiene was not found to be a chemical indicator for any species. In addition, ketones can be produced by the metabolism of sugars (Dillon, 2014). We found that 2,3‐butanedione (diacetyl), a reactive diketone produced from pyruvate, was strongly positively correlated with the decomposition of croaker and mahi‐mahi, and its concentration in mahi‐mahi was significantly higher than in other species. The reason for this difference is not clear. It is likely that diacetyl production is increased with aeration (Dillon, 2014) and mahi‐mahi are mostly found in the surface water. Moreover, mono‐ketones could be gradually derived from hydroperoxides through the splitting of fatty acid chains (Zhou et al., 2020). It was found that 2‐heptanone and 3‐hexanone were not possible chemical indicators of decomposition for any species, while others (2‐butanone, 2‐nonanone, 2‐pentanone, 2‐undecanone, 3‐pentanone, and 3‐methyl‐2‐butanone) could be chemical indicators for certain species. The pathways leading to their formation have been reviewed previously (Kawai & Sakaguchi, 1996; Schulz et al., 2020).

#### 3.5.3 Alcohols

Alcohols have been widely detected along with aldehydes and ketones in various seafood by other researchers (Bai et al., 2019; Duflos et al., 2005; Fratini et al., 2012; Joffraud et al., 2001; Jørgensen & Henrik, 2001). In addition to being a potential chemical indicator of decomposition, delicate flavor of very fresh fish could also be contributed to alcohols (Alasalvar et al., 2005; Lindsay, 1994). Our results suggest that 2‐methyl‐1‐butanol could be a chemical indicator of decomposed yellowfin tuna; 2‐methyl‐1‐propanol could be a chemical indicator of decomposed mahi‐mahi, black tiger shrimp, and sockeye salmon; (*E*)‐2‐penten‐1‐ol could be a chemical indicator of decomposed sockeye salmon; and (*Z*)‐2‐penten‐1‐ol could be a chemical indicator of decomposed red snapper, weakfish, and croaker.

Interestingly, (*Z*)‐2‐penten‐1‐ol, 2‐methyl‐1‐butanol, and 3‐methyl‐1‐butanol showed an obvious downward trend in decomposed weakfish, mahi‐mahi, and sockeye salmon, compared with their fresh counterparts. Similar behavior of volatiles was also observed in other researchers’ data (Duflos et al., 2005; Miyasaki et al., 2011; Zhang et al., 2020). We speculated that it may be attributed to bacterial activities during transportation. For example, the generation of 3‐methyl‐1‐butanol from 3‐methyl‐1‐butyraldehyde is a function of alcohol dehydrogenase (ADH), which catalyzes the interconversion between alcohols and aldehydes (Lu et al., 2012).

#### 3.5.4 Sulfur compounds

Sulfur compounds (dimethyl sulfide, dimethyl disulfide, dimethyl trisulfide, and carbon disulfide) were found to be good indicators of decomposition for different seafood species. This result is in accordance with previous research (Bai et al., 2019; Duflos et al., 2005). The generation of aliphatic sulfur‐containing volatile compounds in seafood has been confirmed to be caused by enzymatic degradation of alk(en)yl cysteine sulfoxides (Varlet & Fernandez, 2010). The development of sulfurous and putrid odors could be the turning point in the spoilage process (Whitfield, 1998), as it can move the overall seafood odor from desirable to rotten. Thus, it would be necessary to monitor the level of sulfur‐containing volatile compounds to better control the quality of seafood. It is interesting that higher concentrations of dimethyl trisulfide (DMTS) and dimethyl disulfide (DMDS) were found in sockeye salmon than in other seafood species. The trend toward production of DMTS and DMDS is consistent with that reported for wild sea bream (Alasalvar et al., 2005), but the cause of accumulation in salmon is currently unknown.

#### 3.5.5 Other target compounds

Ethyl butyrate, an ester having a fruity flavor, was found to be indicative of the quality deterioration of croaker, mahi‐mahi, black tiger shrimp, and sockeye salmon. It has a low odor threshold (1 ppb; Singh & Singh, 2008). *Pseudomonas fragi* may produce ethyl butyrate in fish during the early stages of spoilage (Wang et al., 2017).

Other compounds (alkane, chlorinated hydrocarbon, aromatic hydrocarbons, and saturated hydrocarbons) have been found by other researchers to exist in various seafood products, but their natural sources are poorly understood. Hsieh et al. suggested that benzene derivatives could be present due to environmental pollutants in crayfish (Tanchotikul & Hsieh, 1989). Alasalvar et al. (2005) determined that chloroform did not appear to contribute to the aroma of sea bream and may be an artifact. Heptane, toluene, chloroform, 1,1,3‐trimethylcyclohexane, 1,2‐dimethylcyclohexane, and methylcyclohexane were investigated in the present study. The results indicate that they were generally not reliable chemical indicators for fish spoilage, since their intensities were not strong enough for having sufficient statistical power. Previously, it has been reported that the occurrence of heptane in mackerel could be caused by spoilage (Duflos et al., 2005), however, heptane was not detected in any of the seven seafood species of interest in the current study.

It should be noted that the contribution of specific volatile compound to seafood's off‐odor and off‐flavor is a combined effect of its concentration in seafood and odor detection threshold (Tuckey et al., 2013). In this work, we established a new LVHS‐GC/MS approach to detect the concentration change in various compounds in decomposed and fresh seafood, while their aroma properties have been identified previously by sensory panels. The change in concentration of important markers in decomposed and fresh samples for each species is illustrated in Figure 4. However, we could not determine if a change in the concentration of one specific compound would result in a change in sensory assessment. Extension of this work using the gas chromatography–olfactometry (GC‐O) technique (Brattoli et al., 2013) will be implemented in the future to investigate specific effect of each target compound's concentration change on the sensory evaluation of seafood freshness.

## 4 CONCLUSIONS

This study demonstrated the ability of a large volume headspace GC/MS method for evaluating the quality of seven types of FDA NSSE seafood samples with verified sensory scores. With the completion of this initial study, the LVHS–GC/MS technique showed promising results as a screening tool for differentiating fresh and decomposed seafood samples. The entire process was fully automated and required minimal sample preparation.

Potential marker compounds relating to the quality deterioration were identified and dependent on seafood type. The formation of several aldehydes (2‐methylbutanal, 2‐methyl‐1‐propanal, 3‐methylbutanal, decanal, hexanal, nonanal, and pentanal), ketones (2,3‐butanedione, 2‐butanone, 2‐nonanone, 2‐pentanone, 2‐undecanone, and 3‐methyl‐2‐butanone), alcohols (2‐ethyl‐hexanol, 2‐methyl‐1‐butanol, 2‐methyl‐1‐propanol, 3‐methyl‐1‐butanol, and 2‐penten‐1‐ol), sulfur compounds (dimethyl sulfide, dimethyl disulfide, dimethyl trisulfide, and carbon disulfide), and ethyl butyrate could be proposed as chemical indicators of decomposition for certain species, and especially 3‐methylbutanal, which was significant as a common indicator among all seven seafood types. During these studies, however, it was determined that the LVHS‐GC/MS was not able to differentiate between seafood samples with sensory scores of barely passing (40–49) and barely failing (50–60). Future work will focus on investigating alternative methods to accurately quantify the potential markers identified in this study over an entire sensory sample pack (scores ranging from 10 to 80) to further evaluate the possibility of a rapid analytical method to complement sensory analysis.

## AUTHOR CONTRIBUTIONS

**Zhengfang Wang:** dataCuration (lead) ; formalAnalysis (lead); writingOriginalDraft (lead). **Lowri S. de Jager:** projectAdministration (supporting) ; supervision (supporting); writingReviewEditing (supporting). **Timothy Begley:** projectAdministration (supporting) ; supervision (supporting); writingReviewEditing (supporting). **Susan Genualdi:** methodology (supporting) ; projectAdministration (lead); supervision (lead); writingReviewEditing (lead).

## CONFLICT OF INTEREST

The authors declare no conflict of interest.

## ETHICAL APPROVAL STATEMENT

This study does not involve any human or animal testing.

## Supporting information

Supplementary Material

## Data Availability

The data that support the findings of this study are available on request from the corresponding author.
