# Supplementary material for: Large volume headspace GC/MS analysis for the identification of volatile compounds relating to seafood decomposition
Source: Food Sci Nutr. 2022 Jan 22;10(4):1195–210. doi: 10.1002/fsn3.2751 (PMC9007289; doi:10.1002/fsn3.2751)
Supplement: Supplementary file 1 — Supplementary Material [file FSN3-10-1195-s001.docx]

**SUPPORTING INFORMATION**

**Large Volume Headspace GC/MS Analysis for the Identification of Volatile Compounds Relating to Seafood Decomposition**

Zhengfang Wang ^1^, Lowri S. de Jager ^2^, Timothy Begley ^2^, and Susan Genualdi ^2^^[[1]](#footnote-1)^*

1. Joint Institute for Food Safety and Applied Nutrition, University of Maryland, 2134 Patapsco Building, College Park, MD 20742

2. Center for Food Safety and Applied Nutrition, Office of Regulatory Science, U.S. Food and Drug Administration, College Park, MD 20740

The present study used a large volume headspace sampling method. A seafood sample was grounded and placed in a 1-L headspace vial having an o-ring sealed cap (Figure S1). Volatile compounds accumulated in the gas phase above the ground seafood in the vial. Aliquot of 50 mL vapor in the vial was withdrawn through the valve on the cap.

**Figure S1.** Pictures of (A) a headspace vial lid with the Bottle-Vac™ o-ring seal, (B) a vacuum-packed sockeye filet, and (C) a headspace vial containing analytical sample.

Development and optimization of the large volume headspace sampling method were performed using fresh and unprocessed Atlantic salmon obtained from local supermarkets in the Washington D.C. area. Fillet portions of fresh Atlantic salmon were shredded and spiked with analytical standards at the predicted concentration levels as in decomposed seafood. Aliquots of 50 g spiked salmon were placed in 1-L headspace vials for testing. The incubation temperature was 30 °C throughout the experiments. After incubation, the headspace vials were pressurized to 3.8 psi, the pressure of the GC inlet, using the GC carrier gas (helium). Thus, the total pressure in all vials was uniform. Optimized parameters of the headspace sampling procedures were incubation time (Figure S2), agitation (Figure S3), matrix modifier (Figure S4) and the withdrawn volume of headspace vapor (Figure S5).

**Figure S2.** Effect of the equilibration time on peak areas of compounds eluted at different times. (Blue: incubation time = 30 min; Orange: incubation time = 90 min; Yellow: incubation time = 180 min)

Partitioning is a process that takes a finite time to complete. In many applications, the equilibration time is longer than the GC analysis time. Figure 2S shows that analytes reached equilibration in the headspace vapor after a 30-min incubation at 30 °C.

**Figure S3.** Effect of agitation during incubation (Blue: incubation without shaking; Orange: incubation with active shaking)

Figure S3 shows that the concentrations of analytes in headspace vapor were drastically increased by agitation.

**Figure S4.** Effect of removing water from matrix (Blue: dry matrix; Red: wet matrix)

Figure S4 shows that removing moisture from headspace vapor by adding sodium sulfate, a drying agent, to ground seafood has obviously promoted signals of most compounds.

**Figure S5.** Change of peak areas when different volumes of headspace vapor were withdrawn. (Blue: 10 mL of headspace vapor; Orange: 90 mL of headspace vapor; Yellow: 270 mL of headspace vapor; Purple: 450 mL of headspace vapor)

Figure 5S shows that collecting a large volume of headspace vapor was advantageous for the detection of compounds with medium or low partition coefficients, such as fatty aldehydes in fish oil. However, chromatographic peaks of lighter compounds expanded severely when 90 mL of headspace vapor was injected to GC (data not shown). Moreover, extracting a large volume of headspace vapor undesirably prolonged the overall analysis time. For example, it has taken an extra 44 min, in addition to the 30 min incubation period, to withdraw 450 mL headspace vapor from the sample vial (Table S1). In the current study, the headspace vapor sample size was 50 mL and the recovery of all target compounds was satisfactory.

**Table S1. Headspace Sampling Time**

| **Volume of the extracted headspace vapor (mL)** | **Sampling time (min)** |
| --- | --- |
| 10 | 19 |
| 90 | 24 |
| 270 | 32 |
| 450 | 44 |

A 7650HS-CTS headspace analyzer was used to perform the entire headspace sampling work. A capillary column trapping system (CTS) and a cryo-focuser were used to concentrate sample and focus analytes onto GC column, respectively. Important experimental parameters were listed in Table S2.

**Table S2. Important Capillary Column Trapping System and Cryo-Focuser Parameters**

| **Transfer line Temperature** | 100 °C |
| --- | --- |
| **CTS Trap Temperature (initial)** | 35 °C |
| **CTS Trap Temperature (final)** | 150 °C |
| **CTS Trap Temperature (post-analysis bakeout)** | 150 °C |
| **Trap Flow Rate (nitrogen)** | 20 mL/min |
| **Cryo-Focuser Temperature (focusing)** | -170 °C |
| **Cryo-Focuser Temperature (injection)** | 150 °C |
| **Focusing Time** | 3 min |
| **Post-analysis Bakeout Time** | 10 min |

A Selected Ion Monitoring (SIM) acquisition method was created. Initially, filter paper-based calibration curves were used to assess the established LVHS-GC/MS method. Filter paper spikes were used to evaluate the precision and accuracy of the obtained calibration curves. Results are provided in Table S3. For filter paper spikes, the accuracy and precision of filter-paper based calibration curves have fulfilled expectations, indicating that the established LVHS-GC/MS method has a sound basis for quantitative analysis of volatiles. However, since filter-paper based calibration curves did not encompass matrix effects, quantitative results of cod spikes was not satisfactory. Pooled matrix-matched calibration curves must be used for quantifying target compounds in seafoods. Statistical determination of marker compounds for 7 seafood species is provided in are provided in Table S4.

**Table S3. Linearity, Precision, and Accuracy of Filter Paper Based Calibration Curves**

| **Compounds** | **Filter Paper Based Calibration Curves** | | | | **Filter Paper Spikes** (n=3) | | |
| --- | --- | --- | --- | --- | --- | --- | --- |
|  | **R^2^** | **Lowest Conc.** | **Highest Conc.** | **Spiked Conc.** | **Calculated Conc. (ppb)** | | **Accuracy** |
|  |  | (ppb) | (ppb) | (ppb) | Average | %RSD |  |
| **dimethyl sulfide** | 0.9912 | 104 | 1809 | 1468 | 1273 | 2% | 87% |
| **carbon disulfide** | 0.9965 | 31 | 541 | 439 | 342 | 10% | 78% |
| **2-methyl-1-propanal** | 0.9941 | 190 | 3311 | 2686 | 2425 | 15% | 90% |
| **2,3-butanedione** | 0.9958 | 468 | 8180 | 6637 | 6546 | 16% | 99% |
| **2-butanone** | 0.9922 | 105 | 1831 | 1485 | 1182 | 12% | 80% |
| **chloroform** | 0.9919 | 37 | 642 | 521 | 433 | 12% | 83% |
| **2-methyl-1-propanol** | 0.9854 | 99 | 1731 | 1405 | 1152 | 9% | 82% |
| **3-methylbutanal** | 0.9914 | 193 | 3365 | 2731 | 2482 | 8% | 91% |
| **3-methyl-2-butanone** | 0.9918 | 392 | 6852 | 5560 | 5003 | 8% | 90% |
| **2-methylbutanal** | 0.9907 | 378 | 6600 | 5355 | 5031 | 12% | 94% |
| **2-pentanone** | 0.9909 | 20 | 343 | 278 | 274 | 8% | 98% |
| **pentanal** | 0.9883 | 391 | 6824 | 5537 | 5520 | 8% | 100% |
| **3-pentanone** | 0.9789 | 100 | 1739 | 1411 | 1285 | 8% | 91% |
| **1-penten-3-ol** | 0.9900 | 407 | 7105 | 5765 | 6467 | 8% | 112% |
| **heptane** | 0.9902 | 335 | 5851 | 4748 | 5251 | 7% | 111% |
| **methylcyclohexane** | 0.9912 | 38 | 662 | 537 | 574 | 6% | 107% |
| **dimethyl disulfide** | 0.9901 | 26 | 447 | 363 | 312 | 6% | 86% |
| **3-methyl-1-butanol** | 0.9879 | 394 | 6886 | 5587 | 5920 | 12% | 106% |
| **2-methyl-1-butanol** | 0.9854 | 399 | 6972 | 5657 | 5925 | 14% | 105% |
| **toluene** | 0.9914 | 21 | 372 | 302 | 274 | 6% | 91% |
| **2-penten-1-ol, (E)-** | 0.9843 | 199 | 3476 | 2821 | 2897 | 9% | 103% |
| **3-hexanone** | 0.9895 | 198 | 3451 | 2800 | 2722 | 6% | 97% |
| **hexanal** | 0.9804 | 96 | 1673 | 1357 | 1363 | 10% | 100% |
| **2-penten-1-ol, (Z)-** | 0.9708 | 200 | 3501 | 2841 | 2978 | 3% | 105% |
| **ethyl butyrate** | 0.9904 | 429 | 7485 | 6074 | 6035 | 2% | 99% |
| **1,2-dimethylcyclohexane** | 0.9915 | 189 | 3294 | 2673 | 2670 | 3% | 100% |
| **1,1,3-trimethylcyclohexane** | 0.9901 | 19 | 328 | 266 | 269 | 6% | 101% |
| **2,4-octadiene** | 0.9905 | 192 | 3353 | 2720 | 2451 | 7% | 90% |
| **1-hexanol** | 0.9931 | 200 | 3499 | 2839 | 2610 | 5% | 92% |
| **2-heptanone** | 0.9870 | 20 | 351 | 285 | 269 | 5% | 94% |
| **dimethyl trisulfide** | 0.9895 | 29 | 509 | 413 | 326 | 6% | 79% |
| **octanal** | 0.9902 | 39 | 673 | 546 | 503 | 7% | 92% |
| **2-ethyl-1-hexanol** | 0.9868 | 41 | 716 | 581 | 643 | 14% | 111% |
| **2-nonanone** | 0.9915 | 20 | 353 | 286 | 240 | 11% | 84% |
| **nonanal** | 0.9948 | 40 | 700 | 568 | 483 | 6% | 85% |
| **decanal** | 0.9867 | 40 | 703 | 570 | 584 | 14% | 102% |
| **2-undecanone** | 0.9983 | 40 | 699 | 567 | 502 | 22% | 88% |

| \|  \|  \| \| --- \| --- \| \|  \|  \| \|  \|  \| \|  \|  \| \|  \|  \| \|  \|  \| \|  \|  \| \|  \|  \| \|  \|  \| \| 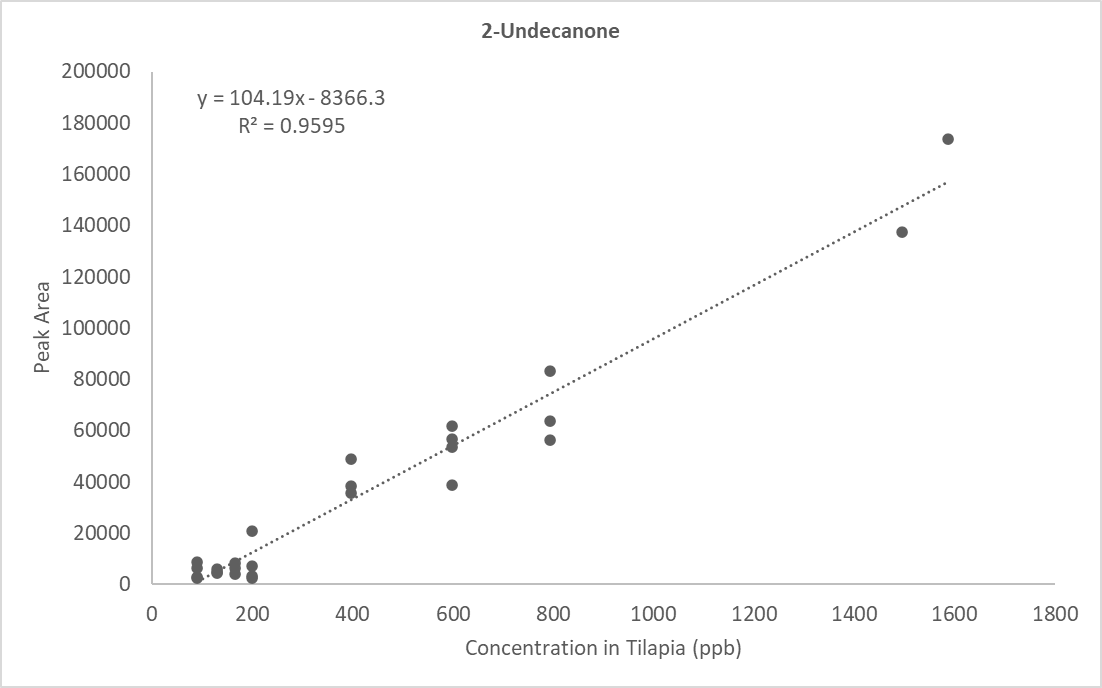 \|  \| \|  \|  \| \|  \|  \| \|  \|  \| \|  \|  \| \|  \|  \| \|  \|  \| \|  \|  \| \|  \|  \| \|  \|  \| \|  \|  \| |
| --- | --- | --- | --- | --- | --- | --- | --- | --- | --- | --- | --- | --- | --- | --- | --- | --- | --- | --- | --- | --- | --- | --- | --- | --- | --- | --- | --- | --- | --- | --- | --- | --- | --- | --- | --- | --- | --- | --- | --- | --- |

**Figure S6.** Pooled matrix-matched calibration curves of 37 target compounds.

Pool matrix-matched calibration curves were obtained by making independent measurements of calibrations at different concentration levels on multiple days. Figure S6 shows pooled matrix-matched calibrations for each compound. The linear regression curve was the line of best fit for the pooled data of all calibrations combined.

**Table S4. Quantitative Determination of Marker Compounds for Seven Seafood Species ^*^**

* Target compounds were quantified using matrix-matched pooled calibration curves provided in the Supplemental Material Figure S6. Sample size (*n*) depended on the availability of the seafood samples under analysis. NQ stands for Not Quantified, meaning the calculated concentration is below the method limit of quantitation. Two-sample t-tests were used to determine whether the averages of “F” (fresh) and “D” (decomposed) groups of samples are significantly different from each other. The *h* value returns a test decision. The result is 1 if the means of two groups is not equal at the 5% significance level (alpha), and 0 otherwise.

1. * Author for correspondence. Tel: (240) 402-2914; E-mail: [Susan.Genualdi@fda.hhs.gov](mailto:Susan.Genualdi@fda.hhs.gov) [↑](#footnote-ref-1)
